# Targeted Discovery of Glycoside Hydrolases from a Switchgrass-Adapted Compost Community

**DOI:** 10.1371/journal.pone.0008812

**Published:** 2010-01-21

**Authors:** Martin Allgaier, Amitha Reddy, Joshua I. Park, Natalia Ivanova, Patrik D'haeseleer, Steve Lowry, Rajat Sapra, Terry C. Hazen, Blake A. Simmons, Jean S. VanderGheynst, Philip Hugenholtz

**Affiliations:** 1 Deconstruction Division, Joint BioEnergy Institute, Emeryville, California, United States of America; 2 Department of Biological and Agricultural Engineering, University of California Davis, Davis, California, United States of America; 3 Department of Energy (DOE) Joint Genome Institute, Walnut Creek, California, United States of America; 4 Microbial Systems Biology Group, Lawrence Livermore National Laboratory, Livermore, California, United States of America; 5 Earth Sciences Division, Lawrence Berkeley National Laboratory, Berkeley, California, United States of America; 6 Biomass Science and Conversion Technology Department, Sandia National Laboratories, Livermore, California, United States of America; Universidad Miguel Hernandez, Spain

## Abstract

Development of cellulosic biofuels from non-food crops is currently an area of intense research interest. Tailoring depolymerizing enzymes to particular feedstocks and pretreatment conditions is one promising avenue of research in this area. Here we added a green-waste compost inoculum to switchgrass (*Panicum virgatum*) and simulated thermophilic composting in a bioreactor to select for a switchgrass-adapted community and to facilitate targeted discovery of glycoside hydrolases. Small-subunit (SSU) rRNA-based community profiles revealed that the microbial community changed dramatically between the initial and switchgrass-adapted compost (SAC) with some bacterial populations being enriched over 20-fold. We obtained 225 Mbp of 454-titanium pyrosequence data from the SAC community and conservatively identified 800 genes encoding glycoside hydrolase domains that were biased toward depolymerizing grass cell wall components. Of these, ∼10% were putative cellulases mostly belonging to families GH5 and GH9. We synthesized two SAC GH9 genes with codon optimization for heterologous expression in *Escherichia coli* and observed activity for one on carboxymethyl cellulose. The active GH9 enzyme has a temperature optimum of 50°C and pH range of 5.5 to 8 consistent with the composting conditions applied. We demonstrate that microbial communities adapt to switchgrass decomposition using simulated composting condition and that full-length genes can be identified from complex metagenomic sequence data, synthesized and expressed resulting in active enzyme.

## Introduction

Enzymatic hydrolysis is one of the most expensive steps in biofuel production from lignocellulosic biomass primarily due to the need for high enzyme loading caused by low catalytic efficiencies [1], [2]. Microorganisms including bacteria and fungi are well-known plant biomass decomposers in nature making them attractive targets for enzyme discovery. Since a variety of biomass sources are envisioned for future biofuel production (e.g. switchgrass, miscanthus, poplar), a broad spectrum of lignocellulolytic enzymes (cellulases, hemicellulases, ligninases) is required to meet future demands. These enzymes are highly modular and usually classified by their domain structure [3]. Glycoside hydrolases (GHs) are a prominent group of enzymes that hydrolyze the glycosidic bond between carbohydrate molecules. The GH families 5, 7 and 9 are the most diverse of the 115 currently recognized GH families, and are of great interest for industrial applications due to their plant cell wall depolymerizing activities [4]. Despite extensive efforts to engineer existing glycoside hydrolases to improve activity and stability, there is still a great need to expand the current enzyme repertoire as well as improve our understanding of how these enzymes function in complex environments [5].

In the present study, we incubated compost-inoculated switchgrass under high-solids and thermophilic conditions to facilitate the enrichment of switchgrass-adapted organisms and associated lignocellulolytic enzymes using a sequencing-based metagenomic approach. Composting is a very dynamic high-solids decomposition process in which microorganisms break down organic matter into carbon dioxide, water, and stable humus-like materials throughout mesophilic and thermophilic phases [6]. Therefore, compost microbial communities can tolerate large changes in temperature, redox conditions, and water activity, recovering quickly from major environmental perturbations. This adaptation to extremes in operating conditions suggests the potential for discovering robust lignocellulolytic enzymes that will also tolerate harsh pretreatment approaches under industry-relevant production standards (e.g. dilute acid, ionic liquid, ammonia fiber expansion).

## Results

**Figure 1:**
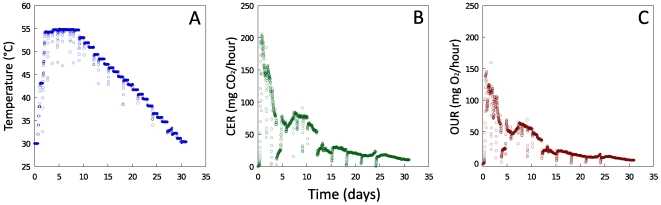
Temperature and respiration profiles during switchgrass incubation. **A,** temperature; **B,** carbon dioxide evolution rates (CER); **C,** oxygen uptake rates (OUR). Brief drops in temperature, CER and OUR levels every three days indicate mixing and water addition.

The bioreactors were established using a switchgrass feedstock inoculated with green-waste compost at a ratio of 9∶1. During a 31-day incubation period, temperature was controlled to simulate a typical composting process: the temperature was maintained at 30°C for 14 hours to allow compost microorganisms to establish, then the temperature was increased from 30°C to 54°C over the course of two days to simulate the self-heating phase, maintained at 54°C for 7 days to simulate the thermophilic phase and slowly decreased back to 30°C over the course of the remaining 21 days to simulate the cooling and maturation phase (Figure 1A). Carbon dioxide evolution (CER) and oxygen uptake rate (OUR) were calculated from continuous measurements of carbon dioxide and oxygen. Both CER and OUR peaked after one day of composting corresponding to initial consumption of sugars [7] and again after eight days during the thermophilic phase (Figure 1B & 1C). This second peak in respiration corresponds to the increased activity of thermophiles [8]. Respiration rates decreased by the end of the incubation, but microorganisms were still active indicating that substrate degradation continued. Periodic mixing and water addition resulted in drops in respiration, followed by rapid recovery (Figure 1B & 1C).

The initial and final samples were analyzed for substrate composition. Total solids decreased 34% during the 31-day incubation. Furthermore, total lignin decreased 17% and sugars associated with hemicellulose and cellulose decreased 28% on average, with the exception of mannose, which was present in low levels in the initial sample and was not detected in the final sample (Table 1).

**Table 1:** Substrate analysis of initial and final sample.

	Initial (g/kg switchgrass + compost)	Final (g/kg switchgrass + compost)^a^	Loss after incubation (%)
Acid-soluble lignin	64	23	64
Acid-insoluble lignin	250	237	5
*Total lignin*	*314*	*260*	*17*
Glucose	260	191	27
Xylose	170	118	31
Galactose	18	12	33
Arabinose	23	16	30
Mannose	0.4	0	100

a Final composition is presented assuming there was no depletion of ash during incubation. Initial and final ash contents were 11.5% and 17.5% of the total dry solids, respectively.

### Microbial Community Composition and Dynamics

**Figure 2:**
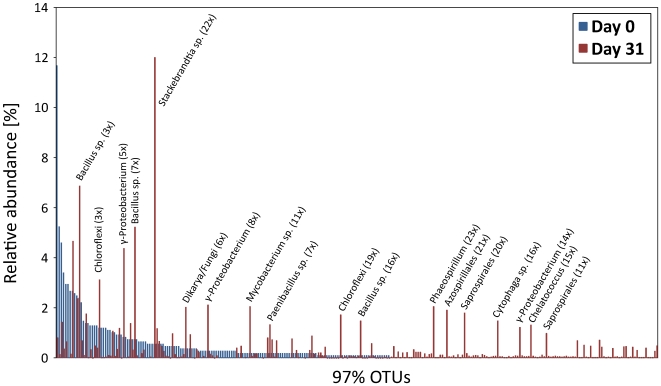
Rank abundance profiles of SSU rRNA phylotypes identified in the initial (day 0) and final (day 31) samples of the compost bioreactor. Overlapping bars indicate the same taxa.

Microbial community structure was determined for the initial (day 0) and final (day 31) bioreactor sample and for a sample of the compost inoculum using small-subunit (SSU) rRNA gene amplicon pyrosequencing. The day 0 sample had a similar microbial community structure to the inoculum (Bray-Curtis dissimilarity 0.501), with the exception that the day 0 community was dominated by switchgrass sequences (69.7% nuclear and 8.2% chloroplast). This suggests that the indigenous microbiota on the switchgrass contributed negligibly to the microbial biomass in the day 0 system. By day 31, the microbial community profile had no discernable correlation to the day 0 profile (Bray-Curtis dissimilarity 0.854) suggesting adaptation of the compost community to the switchgrass feedstock. Also, switchgrass sequences were drastically reduced in the day 31 sample (0.2% nuclear and chloroplast) suggesting that at least degradation of the switchgrass DNA had occurred. Figure 2 shows the rank abundance of the day 0 sample phylotypes overlaid with the day 31 phylotypes. Numerous phylotypes (labeled in Figure 2) increased over the 31-day incubation with up to 23-fold enrichments in relative abundance, including taxa that were below the detection threshold (0.09% populations) in the initial sample (Figure 2). The dominant phylotype in the day 31 sample is closely related to the actinobacterial genus, *Stackebrandtia*, a member of which, *S. nassauensis* (acc. no. NZ_ABUS00000000) contains genes encoding cellulases and hemicellulases (Table 2).

**Table 2:** Inventory of putative glycoside hydrolases (GHs) identified in the SAC microbiome.

CAZy family	known activity	pfam domain	SAC	Cow rumen*	Termite hindgut^[18]^	*Stackebrandtia nassauensis* **
**Cellulases**						
GH5	cellulase	PF00150	3.2	1.0	16.3	5.7
GH6	endoglucanase	PF01341	2.1	0	0	5.7
GH7	endoglucanase	PF00840	0.1	0	0	0
GH9	endoglucanase	PF00759	4.3	0.9	3.9	0
GH44	endoglucanase	NA	0.4	0	1.0	0
GH45	endoglucanase	PF02015	0	0	0.6	0
GH48	endo-processive cellulases	PF02011	0.5	0	0	0
**Total**			**10.6**	**1.9**	**21.8**	**11.4**
**Endohemicellulases**						
GH8	endo-xylanases	PF02011	0.5	0.6	2.7	0
GH10	endo-1,4-β-xylanase	PF00331	8.9	1.0	12.1	0
GH11	xylanase	PF00457	1.4	0.1	2.5	0
GH12	endoglucanase & xyloglucan hydrolysis	PF01670	0.6	0	0	0
GH26	β-mannanase & xylanase	PF02156	1.5	0.7	2.8	2.9
GH28	galacturonases	PF00295	0.9	0.7	1.7	0
GH53	endo-1,4-β-galactanase	PF07745	0.2	2.5	2.7	0
**Total**			**14**	**5.6**	**24.5**	**2.9**
**Cell wall elongation**						
GH16	xyloglucanases & xyloglycosyltransferases	PF00722	2.0	0	0.3	11.4
GH17	1,3-β-glucosidases	PF00332	0.1	0	0	0
GH74	endoglucanases & xyloglucanases	NA	1.6	0	1.0	0
GH81	1,3-β-glucanase	PF03639	0.3	0	0	0
**Total**			**4**	**0**	**1.3**	**11.4**
**Debranching enzymes**						
GH51	α-L-arabinofuranosidase	NA	7.8	9.9	3.5	1
GH54	α-L-arabinofuranosidase	PF09206	0	0.2	0	0
GH62	α-L-arabinofuranosidase	PF03664	1.7	0	0	0
GH67	α-glucuronidase	PF07477, PF07488	3.6	0	2.3	0
GH78	α-L-rhamnosidase	PF05592	8.1	5.1	0.9	2.9
**Total**			**21.2**	**15.2**	**6.7**	**2.9**
**Oligosaccharide-degrading enzymes**						
GH1	β-glucosidase and many other β-linked dimers	PF00232	9.2	1.5	3.1	5.7
GH2	β-galactosidases and other β-linked dimers	PF02836, PF00703, PF02837	8.6	28.6	8.8	2.9
GH3	mainly β-glucosidases	PF00933	12.2	27.0	12.8	17.1
GH29	α-L-fucosidase	PF01120	2.1	4.2	0	0
GH35	β-galactosidase	PF01301	0.6	1.8	0.7	0
GH38	α-mannosidase	PF01074, PF07748	2.6	2.6	5.6	5.7
GH39	β-xylosidase	PF01229	1.0	0.3	1.3	0
GH42	β-galactosidase	PF02449, PF08533, PF08532	2.5	1.7	4.8	22.9
GH43	arabinases & xylosidases	PF04616	11.3	9.4	8.3	17.1
GH52	β-xylosidase	PF03512	0	0	0.4	0
**Total**			**50.1**	**77.1**	**45.8**	**71.4**
**Total GHs**			**801**	**651**	**653**	**35**
**%GHs in total ORFs**			**0.72**	**0.78**	**0.78**	**0.54**

GHs are grouped according to major functional role and compared to other lignocellulosic systems. Both partial and full-length sequences are included. The indicated values are percentages weighted according to species abundance distribution, meaning that the contribution of dominant populations is larger than rare populations.

*indicated numbers are average values of the four cow rumen metagenome data sets published in [19].

**Stackebrandtia nassauensis LLR-40K-21, DSM 44728 (GenBank acc. no. NZ_ABUS00000000).

NA: no pfam domains available for these GH families.

### Metagenome Analysis

To investigate the diversity of genes encoding glycoside hydrolases in the switchgrass-adapted compost (SAC) community, we shotgun sequenced DNA extracted from the day 31 sample using 454-titanium technology. Metagenome sequencing resulted in 548,733 reads with an average read length of 432±108 bp totaling 225 Mbp of sequence data. A considerable proportion of the reads could be assembled into contigs ≥1 kb (a total of 8,268 contigs) with the largest contig of 49,537 bp. This contig is circular (and therefore complete) and encodes 84 putative genes, of which 27% have a highest match to, and shared gene order with, the genome of a novel circular virus, Iodobacteriophage (NCBI acc. no. NC_011142), including capsid, baseplate and tail fiber proteins (Figure S1). Interestingly, this virus also encodes a putative family 43 glycoside hydrolase with 43% similarity to an arabinosidase from the fungus *Armillariella tabescens*.

To compare global functional content of the SAC community metagenome to other lignocellulosic habitats and to non-cellulosic habitats, we performed a correspondence analysis using SEED [9] annotation. The SAC metagenome did not cluster with other lignocellulosic systems (Figure S2), and indeed the SAC community was most closely related to non-lignocellulosic systems including a hypersaline mat, whalefall and a phosphorus-removing bioreactor community. This suggests that genes involved in or associated with lignocellulose metabolism do not contribute enough functional signal in this type of global analysis to cluster lignocellulosic systems together.

Lignocellulosic enzymes were identified by pfam HMMs and grouped according to major functional role (Table 2). Like other communities adapted to lignocellulose degradation, the SAC community had >0.5% of its genes involved in cellulose and hemicellulose deconstruction. Of these genes, 10.6% were putative cellulases, mainly belonging to glycoside hydrolase families GH5 and GH9. This relative abundance of cellulases in the SAC community was ∼5-fold higher than in cow rumen but only half of that in a termite hindgut community. Similar to the cow rumen, a high proportion of carbohydrate-active enzymes found in the SAC community are involved in hemicellulose degradation, particularly in side chain processing (debranching and cell wall elongation enzymes), that may reflect the common substrate type – grass – degraded in these ecosystems. Consistent with this inference is the enrichment of GH families likely involved in depolymerization of the major grass hemicellulose, glucoronoarabinoxylan [10], in the SAC community. This includes putative α-arabinofuranosidases (GH51 and 62) that cleave off arabinose side chains, α-glucoronidases (GH67) that remove glucoronic acid side chains and xylanases (GH10 and 11) that would break down the xylan backbone of glucoronoarabinoxylan (Table 2). Additionally, putative α-rhamnosidases (GH78) were enriched in the SAC and rumen communities relative to the drywood-eating termite hindgut microbiome, although the main rhamnose-containing heteropolysaccharide, pectin, is more prevalent in dicot cell walls than grass cell walls [10]. Oligosaccharide processing enzymes reflect to some degree the inferred polymer breakdown. For example, putative β-xylosidases or β-arabinosidases (GH43) that are involved in breakdown of glucoronoarabinoxylan oligosaccharides are enriched in the SAC community (Table 2).

Complete genes are desirable for enzyme characterization but difficult to obtain from highly fragmented metagenomic datasets [11], such as the SAC metagenome. After frame-shift corrections (an artifact of 454-titanium data), we identified 25 candidate enzymes with a significant match to characterized cellulases or hemicellulases in the CAZy database [3], Table S1). These include two divergent GH9 representatives (36% amino acid similarity to each other) that are most closely related to members of the Alphaproteobacteria (gene JMC20181_1; 68% similarity) and Actinobacteria (gene JMC00312_1; 84% similarity) (Figure S3).

### Cellulase Characterization

**Figure 3:**
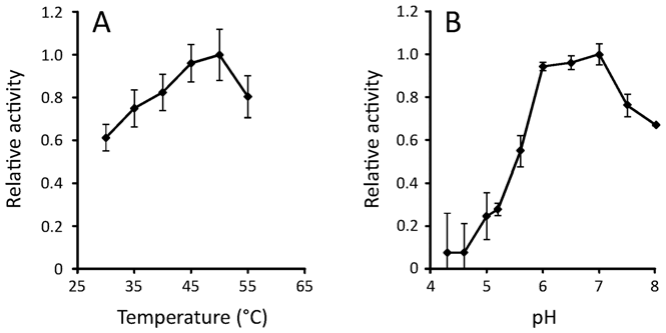
Temperature and pH profiles of the GH9 cellulase JMC00312_1. Soluble extracts from *E. coli* were used to determine temperature (**A**) and pH (**B**) profiles of the heterologous expressed enzyme.

The two full-length GH9 catalytic domains were synthesized (GenScript, Piscataway, NJ) and codon-optimized for expression in *E. coli*. Protein expression was detected for both enzymes with much lower amounts of JMC00312_1 being produced. Soluble extracts of both enzymes were used to test for enzymatic activity on carboxymethyl-cellulose (CMC), 4-nitrophenyl-β-D-cellobioside (pNPC) and 4-nitrophenyl-β-D-glucopyranoside (pNPG). Activity on CMC was only detected for JMC00312_1 despite its low expression level and neither enzyme was active on pNPC or pNPG (Table S2) suggesting that JMC00312_1 is an endoglucanase lacking cellobiosidase or β-glucosidase activity. Furthermore, we suspect that JMC00312_1 is truncated at the C-terminal because its closest homolog (76% similarity) is a much longer actinobacterial endo-/exocellulase (ZP_04475820.1) comprising a conserved domain arrangement; GH9-CBM2-fn3-CBM3. These additional domains would likely enhance endoglucanase activity of the enzyme or may confer endo-/exoglucanase activity to the compost-derived enzyme. Soluble extracts of *E. coli* containing over-expressed enzyme were used to determine temperature and pH profiles of the JMC00312_1 cellulase. The enzyme had an activity optimum of 50°C and pH 7, but retained >50% of its optimum activity over a range of temperatures (30 to 55°C) and pH (5.5 to 8) (Figure 3).

## Discussion

The current dependency on fossil fuels for transportation has put remarkable focus on sources of alternative renewable liquid transportation fuels in recent years [12], [13], [14]. Much of the current research in this area is focusing on so-called second-generation biofuels made from cellulosic biomass of non-food crops. Switchgrass is one of the leading feedstock candidates (others are miscanthus and sorghum) for biofuel production [15], [16], [17]. The goal of this study was to establish a switchgrass-adapted compost (SAC) community using simulated composting conditions in order to select for enzymes capable of degrading switchgrass lignocellulose. We chose to use random shotgun sequencing (metagenomics) for enzyme discovery, an approach that has been successfully used to mine other lignocellulosic ecosystems for plant cell wall depolymerizing enzymes (e.g. termite hindgut [18], cow rumen [19]).

Over the course of a 31-day composting experiment, measurements of reduction in solids, sugar content (Table 1) and O_2_ uptake and CO_2_ evolution rates (Figure 1) indicated active degradation of the switchgrass biomass. In a study examining the decomposition of *Miscanthus* straw, 10–20% degradation of both cellulose and hemicellulose was observed during the first three months of composting [20]. During composting of ryegrass straw, lignin loss measured using the Klason method was 15% over a 30-day period [21]. Comparable decomposition levels for lignin (17% loss in total lignin) and cellulose and hemicellulose (28%) in this study (Table 1) indicate that the bioreactor management approach sufficiently simulated an environment that might be encountered in a straw-based composting process. Switchgrass can be effectively degraded by microorganisms as shown in a previous study investigating deconstruction of the leaf blade, leaf sheath and stem of this species by cow rumen communities [22]. Results from microcosm studies in which stems and leaves from switchgrass (Sunburst) were separated, incorporated into soil and incubated at 25°C for 498 days also demonstrated that leaves and stems will decompose in a high-solids soil environment [23].

Microbial community composition changed dramatically between the initial and final bioreactor sample (Figure 2) suggesting selection of specific populations to degrade the switchgrass biomass. Composting is a highly dynamic process selecting for different species during the various composting stages [6], [24], [25]. Mesophilic bacteria and fungi dominate the initial composting microbial communities utilizing the soluble and easily metabolized carbohydrates from the fresh organic substrates [7]. During the subsequent thermophilic phase, Actinobacteria feeding on recalcitrant plant cell wall components dominate communities [26], [27]. Consistent with these generalized compost observations, we noted an increase in Actinobacteria from 16% to 23% between the initial and switchgrass-adapted communities. Moreover, the dominant population in the SAC community, enriched 22-fold from the initial sample (Figure 2), was an actinobacterium related to the genus *Stackebrandtia*. From only two time points, we cannot tell when the *Stackebrandtia*-like population became enriched, i.e. during the thermophilic or cooling and maturation phase.

Adaptation of the compost microbial community to the switchgrass biomass is reflected in the number of glycoside hydrolases identified in the metagenomic dataset which account for >0.5% of all genes called (Table 2). This is characteristic of ecosystems that have evolved to degrade lignocellulosic substrates [18], [19]. Unlike dicots, the major hemicellulose in grass cell walls is glucoronoarabinoxylan composed of a β-1,4-linked xylose backbone with single arabinose and glucoronic acid side chains [10]. We identified a high proportion of genes encoding enzymes that are likely to degrade this type of hemicellulose including debranching GH families specific to the arabinose and glucoronic acid side chains (GH51, 62, 67). These same families are present in much lower proportions in the drywood-feeding termite hindgut (Table 2). Conversely, the termite hindgut microbiome has a higher proportion of cellulases than the SAC and rumen communities possibly reflecting the typically higher cellulose content in dicots than grasses [10].

Of the putative cellulases identified in the SAC community, the highest proportion belongs to glycoside hydrolase family 9. Enzymes of the GH9 family also can act as 1,4-β-cellobiosidases or β–glucosidases (www.cazy.org, [3]) and have been found in a variety of ecosystems including insects [18], [28], cow rumen [19] and human distal gut [29]. We synthesized two full-length GH9 domains from the metagenomic data with codon optimization for expression in *E. coli* and demonstrate that one has endoglucanase but not cellobiosidase or β–glucosidase activity. The active cellulase, JMC00312_1, is possibly of actinobacterial origin due to its association with other actinobacterial sequences (Figure S3). This is further supported by temperature and pH profiles of the enzyme (Figure 3) suggesting that it is functional under thermophilic and slightly alkaline conditions characteristic of the thermophilic composting phase, which typically selects for Actinobacteria [6]. Based on these findings we anticipate that the combination of composting conditions (30–55°C, pH 6–8), use of a targeted feedstock and codon optimization of identified candidate enzymes to improve heterologous expression will supply physiologically versatile and feedstock-specific enzymes applicable to emerging pretreatment practices such as ionic-liquid pretreatment [30].

## Materials and Methods

### Bioreactor Inoculation and Operation

A compost inoculum was obtained on August 6, 2007 from a Grover Soil Solutions compost facility located in Zamora, CA. This facility composts green waste and agricultural wastes (e.g. prunings from perennial crops and hulls from nut and rice processing) in turned and watered windrows. Prior to collection the compost had been passed through a trommel screen to remove large debris. Immediately upon collection compost was returned to the laboratory and screened to 3.2 mm. The compost was then solar-dried for 48 hours and stored at 4°C. At the time of the experiment the compost had been stored for approximately five months. The plant biomass was harvested from 2-year old plants of the cultivar Kanlow grown in 6″ pots in a greenhouse. Greenhouse conditions were 75°F and minimum 12-hour day length; plants were watered daily and fertilized monthly with 1 g of fertilizer per pot. Harvested switchgrass was oven-dried at 50°C for five days, milled with a knife mill and passed through a 2 mm screen. Processed material was stored in a sealed container at room temperature until experimentation.

Switchgrass was wetted with distilled water to a target moisture content of 200% on a dry basis and placed at 4°C overnight to allow water and feedstock equilibration. Switchgrass and compost mixtures were prepared with 90% switchgrass and 10% dried compost on a dry weight basis immediately before loading the reactors. Microbial activity studies were conducted as previously described [31] with the following modifications. Reactors with a 0.9 L working volume were filled with 75 dry grams of the switchgrass and compost mixture. Three reactors were connected in series to simulate the oxygen gradient of a compost pile. Reactors were aerated continuously with humidified air at 30 mL min^−1^ and incubated for 31 days.

The temperature of the incubator was controlled to simulate a typical composting process and monitored continuously with a thermocouple connected to a 21x data logger (Campbell Scientific, Logan, UT). Oxygen concentration was measured on the influent and effluent air of the reactors using Zirconia oxide oxygen sensors (Neuwghent Technologies, LaGrangeville, NY) and carbon dioxide concentration was measured using an infrared CO_2_ sensor (Vaisala, Woburn, MA). Oxygen and carbon dioxide data were recorded every 20 minutes using a data acquisition system [31]. Carbon dioxide evolution rates (CER) and oxygen uptake rates (OUR) were calculated from mass balances on the reactors according to the following equations:
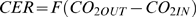


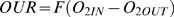
where *F* is the air flow rate (mg air day^−1^ gdw^−1^), CO_2,OUT_ and CO_2,IN_ are the concentrations of carbon dioxide in the effluent and influent air, respectively (mg CO_2_ mg air^−1^), and O_2,IN_ and O_2,OUT_ are the concentrations of oxygen in the influent and effluent air, respectively (mg O_2_ mg air^−1^). JMP v.7 (SAS Institute Inc. Cary, NC) statistical software was used to perform statistical comparisons. Numerical integration of CER and OUR results was performed using KaleidaGraph v. 4.0 (Synergy Software, Reading, PA).

### Biomass Composition Analysis

Moisture content was measured gravimetrically after drying samples at 105°C for 24 hours. Acid insoluble, soluble lignin and carbohydrate content of initial and final mixed samples were determined by adapted NREL CAT Task Laboratory Analytical Procedures #003 [32], #004 [33] and #002 [34], respectively. Precisely weighed 0.5±0.1 mg samples were air-dried at 45°C and suspended in 5 mL of 72% (w/w) H_2_SO_4_ in 200 mL serum bottles. Samples were hydrolyzed at 30°C for 2 hours with agitation every 30 minutes. After hydrolysis the contents of each bottle were diluted to a 4% acid concentration with 140 mL of distilled water. The sample bottles were sealed and autoclaved at 121°C and 21 psi for 1 hour. Cooled samples were vacuum filtered through pre-weighed 2 µm glass fiber filters (Fisher Scientific, Pittsburgh, PA). The filtrate was sampled for acid soluble lignin and carbohydrate analysis. Acid soluble lignin was measured by absorbance at 205 nm using 4% H_2_SO_4_ (w/w) as the blank. Monosaccharide content of the filtrate was determined using an HPLC equipped with a Bio-Rad Aminex HPX-87P chromatography column and de-ashing guard cartridge. Samples were passed through 0.2 mm PTFE syringe filters directly into 2 mL sample vials. Samples were injected by autosampler and analyzed at 85°C with sterile-filtered and degassed distilled water as the mobile phase at 0.6 mL/min. The filters were washed with water to remove residual acid and dried in aluminum dishes at 105°C to a consistent weight. The filters were then ignited by increasing the temperature of the furnace at a rate of 10°C min^−1^ and then holding the sample at 550±25°C for 4 hours. Acid-insoluble lignin was determined gravimetrically.

### Nucleic Acid Extraction

Samples for DNA extraction were stored at −80°C. Before extraction, samples were homogenized repeatedly using a TissueLyser (Qiagen, Inc., Valencia, CA) for 30 seconds at 27.7 Hz until uniform particle size was achieved. Prior to each homogenization jars holding the samples were frozen in liquid nitrogen. Approximately 0.5 g of homogenized material was loaded into bead-beating tubes (Lysing Matrix E; MP Biomedicals Life Sciences Division, Solon, OH) and extracted by adding CTAB buffer (equal volumes 10% hexadecyltrimethylammonium bromide in 1M NaCl and 0.5 M phosphate buffer, pH 8 in 1 M NaCl), 0.1 M ammonium aluminum sulfate, and phenol∶chloroform∶isoamylalcohol (25∶24∶1) followed by bead-beating for 30 seconds at 5.5 m/s [35]. This extraction was repeated two times and the aqueous phases of both steps were further purified using chloroform∶isoamylalcohol (24∶1) followed by precipitation of the nucleic acids in 30% PEG 6000 (30% wt/vol Poly(ethylene glycol) 6,000 in 1.6 M NaCl). DNA pellets were washed in 70% ethanol and resuspended in nuclease-free Tris-EDTA buffer.

### Community Profiling

Small subunit (SSU) rRNA gene sequences were amplified using the primer pair 926f/1392r as described in Kunin et al. [36]. The reverse primer included a 5 bp barcode for multiplexing of samples during sequencing. Emulsion PCR and sequencing of the PCR amplicons was performed following manufacturer's instructions for the Roche 454 GS FLX Titanium technology, with the exception that the final dilution was 1e^−8^. Sequencing tags were analyzed using the software tool PyroTagger (http://pyrotagger.jgi-psf.org/) using a 220 bp sequence length threshold.

### Metagenome Sequencing, Assembly and Analysis

Genomic DNA extracted from the day 31 sample was used for sequencing library construction following the DOE Joint Genome Institute standard operating procedure for shotgun sequencing using the Roche 454 GS FLX Titanium technology. Obtained sequencing reads were quality trimmed and assembled using the Newbler assembler software (version 2) by 454 Life Sciences. For assembly, minimum acceptable overlap match (mi) was set to 0.95. Quality filtered sequence reads and assembled contigs ≥100 bp totaling 110 Mbp were used for further analysis. For global functional analysis, the metagenomic data set was loaded into MG-RAST [37] and compared to other annotated metagenomes that are publicly available in the metagenome analysis platform. Correspondence analysis was performed using the R software package ade4 [38].

Glycoside hydrolases of selected functional classes (e.g. cellulases, endohemicellulases, debranching enzymes) were identified using pfam HMMs (Pfam version 23.0 and HMMER v2.3). For the 3 GH families 44, 51 and 74 that are not represented in pfam, HMMs were generated (two for each, since they are 2-domain proteins) and treated similar to the pfam HMMs. For GH families covered by multiple pfams (e.g. GH2 or GH42) only the best scoring hit was taken into account in case there were multiple hits to the same contig. Contig read depth was factored as following: based on the Newbler output, the number of reads in each was determined and multiplied by the median read length of 400 bp and divided by the contig length. This weighting corrects approximately for differences in species abundance distribution (i.e. dominant populations producing higher depth contigs will be weighted in the analysis).

To extract potential full-length glycoside hydrolases from the metagenome data, we ran BLASTX on all contigs ≥1 kb against the CAZy [3] and FOLy [39] databases (E<1e^−10^), and filtered out hits matching the target enzyme over at least 90% of its length, and for which the target enzyme has a known enzymatic function (EC number listed in CAZy or FOLy). Frameshifts (most likely introduced by homopolymers during sequencing) were delineated by BLASTX of the targeted contigs against the non-redundant NCBI nucleotide database and corrected by deleting or duplicating single bases so as to maximize the BLAST scores. After manual frameshift correction, genes were called using fgenesb (http://www.softberry.com). For phylogenetic analysis, peptide sequences of the two full-length GH9 enzymes were aligned to reference sequences using ClustalX [40] and imported into the ARB software package [41] for phylogenetic reconstructions using the PROML function of the integrated Phylip package.

### Cellulase Protein Expression and Activity Screening

The nucleotide sequences of two putative cellulases (contigs JMC00312_1, JMC20181_1) were codon-optimized for protein expression in *E. coli* (GenScript, Piscataway, NJ). The PCR primers were designed to amplify genes without the putative signal peptide sequences (SignalP 3.0 server, http://www.cbs.dtu.dk/services/SignalP/). The amplicons were cloned into the pET DEST42 vector via the Gateway cloning method (Invitrogen, Carlsbad, CA). The plasmids containing the cellulase genes were transformed into the BL21 (DE3) Star strain (Invitrogen, Carlsbad, CA) of *E. coli* for protein expression. Small-scale protein expression was done in 5 mL culture volume for each cellulase gene by auto-induction at 30°C (Overnight Express AutoinductionTM System, Novagen, Gibbstown, NJ). After protein expression, the cells were harvested by centrifugation at 6,000×g for 10 min. The cell lysates were prepared using the BugBuster reagent. The volume of the BugBuster used for each cell pellet was normalized to an OD600 nm reading of the culture (50 µL× OD600 nm). The overnight cultures without protein induction reagent were also prepared for uninduced controls. The cell lysates were centrifuged at 10,000×g for 30 min to separate soluble proteins from insoluble materials. The supernatants (soluble proteins) were collected for SDS-PAGE and enzyme activity screens. To test enzyme activity on carboxymethyl-cellulose (CMC), 2 µL of the supernatant was spotted on an agar plate containing 0.1% CMC. The plate was incubated at 37°C for 2 hours. The enzyme activity was detected by Congo red assay [42]. To determine whether the cellulases have cellobiosidase or β-glucosidase activity, 4-nitrophenyl-β-D-cellobioside (pNPC) or 4-nitrophenyl-β-D-glucopyranoside (pNPG) were used as substrates [43].

Soluble protein extract from *E. coli* containing expressed JMC00312_1 was used to determine temperature and pH activity profiles. All reactions were performed in 50 µL volumes. To measure the pH optimum, a standard pH solutions containing 100 mM sodium acetate, 50 mM MES, and 50 mM HEPES between pH 4 and 8 were used as buffer for the enzyme reaction at 50°C. At the end of a 30 min incubation, 120 µL of DNS reagent [44] was added to the reaction mixture and incubated at 95°C for 5 min to label the reducing ends of hydrolyzed CMC. The absorbance at 540 nm was measured to determine the relative activity across the tested temperature and pH ranges.

### Sequence Data Submission

The raw sequencing reads and the assembled metagenome dataset have been deposited at GenBank and the NCBI Short Read Archive under Genome Project ID 41493 and accession number SRA010300.1, respectively. The SSU rRNA amplicon pyrosequencing reads are deposited under the accession numbers GU178033 - GU178768.

## Supporting Information

Table S1 Putative full-length cellulase and hemicellulase enzyme sequences extracted from the SAC metagenome data set. The two GH9 cellulases in bold were tested for activity on CMC, pNPC and pNPG. (a) Best BLASTX hit against any sequence in CAZy with a validated EC number indicating a lignocellulolytic enzyme. (b) Number of frameshift corrections required, based on alignments with homologs in NR. The prevalence of frameshifts complicates assembly, gene calling, and annotation of genes in low-coverage 454-titanium metagenomic data. For example, we noticed that none of the manually corrected frameshifts for the full-length catalytic domains were caught by the MG-RAST annotation, resulting in truncated genes. (c) Length (in aa) of potential truncation at the N terminal (N) or C terminal (C), due to the end of the contig, based on closest homolog in NR. (d) Contig JMC02101 was originally selected because of a CAZy hit against a GH30 β-xylosidase, but also contains a GH5 endoglucanase gene. *Reference species recently sequenced by the US DOE Joint Genome Institute as part of the Genome Encyclopedia of Bacteria and Archaea.
(0.09 MB DOC)

Table S2 Domain structure, protein expression and activity profiles of the two full-length genes belonging to family GH9. U: uninduced negative control; I: IPTG induced sample.
(0.03 MB DOC)

Figure S1 Chromosome of a circular phage recovered from the SAC community metagenome (contig JMC02169) related to Iodobacteriophage. Genes were predicted using fgenesV (www.softberry.com).
(1.12 MB EPS)

Figure S2 Correspondence analysis of the compost bioreactor microbiome to other lignocellulosic (pentagons) and non-lignocellulosic (circles) microbiomes. The total variance extracted by the coordinate axes was 46.8% (31.1%+15.7%). Metagenome IDs given in the legend correspond to references metagenomes in MG-RAST (http://metagenomics.nmpdr.org/) used for comparative analysis.
(0.09 MB EPS)

Figure S3 Evolutionary distance (maximum likelihood) tree of aligned representatives of glycoside hydrolase family 9 proteins showing the relative position of the two characterized GH9 proteins identified in the SAC microbiome. Bootstrap values (100 bootstraps) are given for selected nodes.
(1.35 MB EPS)
