# Supplementary material for: Targeted Discovery of Glycoside Hydrolases from a Switchgrass-Adapted Compost Community
Source: PLoS One. 2010 Jan 21;5(1):e8812. doi: 10.1371/journal.pone.0008812 (PMC2809096; doi:10.1371/journal.pone.0008812)
Supplement: Table S1 — Putative full-length cellulase and hemicellulase enzyme sequences extracted from the SAC metagenome data set. The two GH9 cellulases in bold were tested for activity on CMC, pNPC and pNPG. (a) Best BLASTX hit against any sequence in CAZy with a validated EC number indicating a lignocellulolytic enzyme. (b) Number of frameshift corrections required, based on alignments with homologs in NR. The prevalence of frameshifts complicates assembly, gene calling, and annotation of genes in low-coverage 454-titanium metagenomic data. For example, we noticed that none of the manually corrected frameshifts for the full-length catalytic domains were caught by the MG-RAST annotation, resulting in truncated genes. (c) Length (in aa) of potential truncation at the N terminal (N) or C terminal (C), due to the end of the contig, based on closest homolog in NR. (d) Contig JMC02101 was originally selected because of a CAZy hit against a GH30 β-xylosidase, but also contains a GH5 endoglucanase gene. *Reference species recently sequenced by the US DOE Joint Genome Institute as part of the Genome Encyclopedia of Bacteria and Archaea. (0.09 MB DOC) [file pone.0008812.s001.doc]

| **SAC**  **Contig** | **Initial BLASTX hit against CAZy ^(a)^** | | | | | **Best BLASTP hit against NR, after frameshift correction and gene calling** | | | | | |
| --- | --- | --- | --- | --- | --- | --- | --- | --- | --- | --- | --- |
|  | **%Id** | **CAZy hit** | **description** | **EC** | **FAM** | **fs^(b)^** | **%Id** | **NR hit** | **description** | **Organism** | **trunc^(c)^** |
| **JMC00312** | **50** | **AAK12339.1** | **endo-1,4-glucanase** | **EC 3.2.1.4** | **GH9** | **0** | **75** | **ZP_04475820.1** | **cellobiohydrolase A (1,4-β-cellobiosidase A)** | ***Streptosporangium roseum***  **DSM 43021^*^** | **283 (C)** |
| JMC00766 | 60 | CAA76421.2 | α-L-arabino-furanosidase | EC 3.2.1.55 | GH51 | 4 | 72 | YP_003121941.1 | α-L-arabinofuranosidase domain protein | *Chitinophaga pinensis*  DSM 2588^*^ | 39 (N) |
| JMC01245 | 79 | CAC19491.1 | xylanase | EC 3.2.1.8 | GH11 | 0 | 68 | ZP_03912958.1 | glycosyl hydrolase family 11 with cellulose-binding domain | *Xylanimonas cellulosilytica*  DSM 15894^*^ | - |
| JMC02101a | 28 | ABC55722.1 ^(d)^ | β-xylosidase | EC 3.2.1.37 | GH30 | 2 | 50 | YP_001983219.1 | cellulase, putative, cel5G | *Cellvibrio japonicus* Ueda107 | - |
| JMC02101b |  |  |  |  |  | 0 | 57 | ZP_01885708.1 | Glucosylceramidase | *Pedobacter* sp. BAL39 | - |
| JMC02364 | 41 | AAC38456.1 | arabino-furanosidase I | EC 3.2.1.55 | GH51 | 4 | 70 | YP_003121941.1 | α-L-arabinofuranosidase domain protein | *Chitinophaga pinensis*  DSM 2588^*^ | - |
| JMC03259 | 38 | AAC98123.1 | xylanase (intracellular) | EC 3.2.1.8 | GH10 | 0 | 54 | ZP_05010737.1 | β-1,4-xylanase | *Streptomyces pristinaespiralis*  ATCC 25486 | 27 (C) |
| JMC04168 | 64 | AAB08024.1 | β-xylosidase/α-L-arabinofuranosidase | EC 3.2.1.37  EC 3.2.1.55 | GH43 | 0 | 72 | YP_526296.1 | Α-L-arabinofuranosidase | *Saccharophagus degradans* 2-40 | - |
| JMC04920 | 72 | EAA59562.1 | α-L-arabino-furanosidase | EC 3.2.1.55 | GH62 | 0 | 77 | ZP_04334946.1 | glycosyl hydrolase family 62 | *Nocardiopsis dassonvillei* subsp. *dassonvillei* DSM 43111^*^ | - |
| JMC04931 | 47 | BAB69073.1 | xylanse F3 | EC 3.2.1.8 | GH10 | 0 | 68 | ZP_05542989.1 | xylanase A | *Streptomyces griseoflavus* Tu4000 | 48 (N)  37 (C) |
| JMC07159 | 46 | AAA96979.1 | xylanase | EC 3.2.1.8 | GH10 | 0 | 52 | YP_001817989.1 | endo-1,4-β-xylanase | *Opitutus terrae* PB90-1 | 282 (N)  362 (C) |
| JMC07447 | 44 | AAZ74783.1 | xylanase | EC 3.2.1.8 | GH10 | 0 | 64 | YP_002881274.1 | Endo-1,4-β-xylanase | *Beutenbergia cavernae*  DSM 12333^*^ | 129 (N)  516 (C) |
| JMC09349 | 30 | AAD35394.1 | α-L-fucosidase | EC 3.2.1.51 | GH29 | 0 | 42 | ZP_01875438.1 | probable glycosyl hydrolase | *Lentisphaera araneosa*  HTCC2155 | - |
| JMC10050 | 43 | AAD35149.1 | α-glucuronidase | EC 3.2.1.139 | GH67 | 2 | 62 | ZP_04422320.1 | α-glucuronidase | *Rhodothermus marinus*  DSM 4252^*^ | 22 (N) |
| JMC10625 | 41 | ABI18350.1 | β-glycosidase | EC 3.2.1.- | GH1 | 0 | 69 | ZP_04497591.1 | broad-specificity cellobiase | *Sphaerobacter thermophilus*  DSM 20745^*^ | 37 (C) |
| JMC10774 | 43 | CAB12521.1 | acetyl xylan- esterase | EC 3.1.1.72 | CE12 | 3 | 46 | YP_001297532.1 | carbohydrate esterase family 12 protein | *Bacteroides vulgatus*  ATCC 8482 | - |
| JMC14576 | 46 | AAZ32298.1 | β-glucosidase | EC 3.2.1.21 | GH3 | 4 | 72 | YP_001195854.1 | glycoside hydrolase family 3 protein | *Flavobacterium johnsoniae*  UW101 | - |
| JMC14824 | 42 | AAK76861.1 | xylanase 10B | EC 3.2.1.8 | GH10 | 2 | 45 | YP_003073132.1 | xylanase | *Teredinibacter turnerae*  T7901 | 211 (C) |
| JMC15193 | 59 | AAC38457.1 | arabino-furanosidase II | EC 3.2.1.55 | GH51 | 0 | 64 | ZP_04771039.1 | Α-N-arabinofuranosidase | *Asticcacaulis excentricus*  CB 48 | - |
| JMC16911 | 44 | CAB12521.1 | acetyl xylan- esterase | EC 3.1.1.72 | CE12 | 3 | 59 | ZP_04493651.1 | lysophospholipase L1-like esterase | *Spirosoma linguale*  DSM 74^*^ | - |
| **JMC20181** | **43** | **AAR87745.1** | **cellobio-hydrolase** | **EC 3.2.1.91** | **GH9** | **1** | **55** | **NP_421030.1** | **glycosyl hydrolase family protein** | ***Caulobacter crescentus***  **CB15** | **-** |
| JMC25406 | 65 | AAB08024.1 | β-xylosidase/α-L-arabino-furanosidase | EC 3.2.1.37  EC 3.2.1.55 | GH43 | 0 | 78 | YP_001981807.1 | β-xylosidase/α-L-arabinfuranosidase, putative | *Cellvibrio japonicus*  Ueda107 | - |
| JMC35591 | 53 | BAB07402.1 | β-xylosidase | EC 3.2.1.37 | GH43 | 0 | 72 | YP_001615527.1 | xylan 1,4-β-xylosidase | *Sorangium cellulosum*  'So ce 56' | 48 (N) |
| JMC37744 | 38 | AAL06078.1 | β-1,4-xylanase | EC 3.2.1.8 | GH10 | 0 | 46 | ZP_04771042.1 | Endo-1,4-β-xylanase | *Asticcacaulis excentricus*  CB 48 | - |
| JMC44805 | 27 | AAA23063.1 | β-xylosidase B | EC 3.2.1.37 | GH39 | 0 | 66 | ZP_05060059.1 | Glycosyl hydrolases family 39 | Verrucomicrobiae bacterium  DG1235 | 28 (C) |
