# Supplementary material for: Targeted Discovery of Glycoside Hydrolases from a Switchgrass-Adapted Compost Community
Source: PLoS One. 2010 Jan 21;5(1):e8812. doi: 10.1371/journal.pone.0008812 (PMC2809096; doi:10.1371/journal.pone.0008812)
Supplement: Table S2 — Domain structure, protein expression and activity profiles of the two full-length genes belonging to family GH9. U: uninduced negative control; I: IPTG induced sample. (0.03 MB DOC) [file pone.0008812.s002.doc]

| Contig identifier | Domain structure | Est. MW (kDa) | Expr in cell lysate | Expr. in soluble fraction | Activity on CMC | | pNPC | pNPG |
| --- | --- | --- | --- | --- | --- | --- | --- | --- |
|  |  |  |  |  | U | I |  |  |
| JMC00312_1 | GH9 | 60 | (+) | - | - | + | - | - |
| JMC20181_1 | celD_N/GH9 | 69 | + | - | - | - | - | - |
